# Hyperreactio luteinalis associated with fetal hyperandrogenism and cystic hygroma

**DOI:** 10.1002/ccr3.6310

**Published:** 2022-09-08

**Authors:** Ali Ghassa, Dema Adwan, Mhd Firas Safadi

**Affiliations:** ^1^ Faculty of Medicine Damascus University Damascus Syria; ^2^ Department of Obstetrics and Gynaecology Maternity University Hospital Damascus Syria; ^3^ Department of General Surgery, Visceral Surgery, and Proctology Diakonie Hospital Hartmannsdorf Germany

**Keywords:** cystic hygroma, Hyperreactio luteinalis, ovarian cysts, pregnancy

## Abstract

A 23‐year‐old woman with a gestational age of 17 weeks presented with abdominal pain. The ultrasound showed maternal hyperreactio luteinalis with fetal cystic hygroma. After termination of pregnancy, the female fetus showed masculinization features with muscular hypertrophy. The hyperreactio luteinalis regressed under hormonal suppression therapy.

## Abstract 2

Hyperreactio luteinalis can develop in spontaneous pregnancies with normal gonadotropin levels. Resolution with conservative therapy is the rule in most cases. This abnormality was accompanied with fetal cystic hygroma in two documented cases.

## 1 INTRODUCTION

Hyperreactio luteinalis is a rare benign condition of pregnancy that is characterized by the formation of multiple large ovarian cysts due to hyperstimulation of the ovarian tissue.^1^ Although the exact causes are unknown, this entity is attributed to elevated levels of human chorionic gonadotropin (hCG) or ovarian hypersensitivity to this hormone.^2^, ^3^ This disease can be associated with other hormonal derangements such as thyroid hormone abnormalities or hyperandrogenism.^4^

The disease may also afflict the fetus, causing intrauterine growth restriction and inducing preterm labor. An association with cystic hygroma was reported in only one case so far.^5^ Fetal cystic hygromas are congenital malformations of the lymphatic system that appear as fluid‐filled cavities and may be associated with chromosomal abnormalities.^6^ With the availability of modern ultrasound devices, prenatal detection is possible in most cases.^7^

In this article, we report a case of hyperreactio luteinalis that presented in the second trimester of spontaneous pregnancy and was associated with fetal cystic hygroma and masculinization of the female fetus. To our knowledge, this is the second documented case of cystic hygroma accompanying maternal hyperreactio luteinalis.

## 2 CASE HISTORY

A 23‐year‐old pregnant woman presented to the emergency department with acute abdominal pain. The symptoms started a few weeks ago and gradually increased in intensity. The pain was described as “heaviness and pressure” in the lower abdomen with occasional radiation to the back. No other gastrointestinal complaints were reported. Additionally, the patient was in her third pregnancy (gravida 3 para 2) with a gestational age of about 17 weeks. The pregnancy showed normal progress so far, but a pregnancy assessment at 12 weeks was missed due to compliance issues. Vaginal bleeding or discharge was denied.

The clinical examination showed stable vital signs with no fever. The palpation revealed a soft abdomen with slight hypogastric tenderness but no guarding or rigidity. The fundal height was at the umbilical level, which slightly exceeded the reported gestational age. No abnormalities were found on vaginal inspection.

**FIGURE 1:**
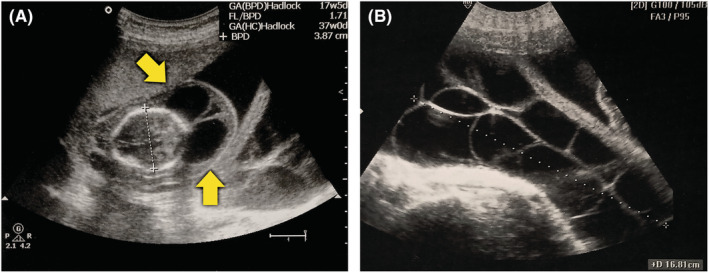
Transabdominal ultrasound examination. (A) Axial view of the fetus showing a cephalic cystic hygroma with two oval‐shaped fluid chambers that lie between the skull and the scalp (arrows). The biparietal diameter equals 3.87 cm. (B) Transverse suprapubic view of the mother showing multiple cystic formations in the right ovary with regular walls and anechoic content. The maximal diameter of the complex equals about 17 cm.

The transabdominal and transvaginal ultrasound showed a single live fetus, normal amniotic fluid, and normal placental position. Based on the biparietal diameter, the estimated fetal age was 17 weeks 5 days. Additionally, the fetal study showed thickened nuchal soft tissues with a cephalic cystic formation measuring 3.5 cm in maximal diameter (Figure 1A). The examination of the maternal abdominal organs showed a bilateral multilocular cystic formation that fills the whole pelvis, which presents a typical appearance of hyperreactio luteinalis (Figure 1B). To complete the study and exclude malignant conditions, we ordered a full laboratory profile including tumor markers and β‐hCG. The results showed gestational anemia and mild elevation of cancer antigen 125 (CA‐125) (Table 1).

**TABLE 1:** Laboratory results

Laboratory assay	Patient's results	Reference range
Hemoglobin	8.1	12–16 g/dl
Hematocrit	23	36%–48%
White blood count	7.9	4.5–11.0 × 10^9^/L
Platelet count	359	150–400 × 10^9^/L
Glucose	68	70–110 mg/dl
Blood urea nitrogen	18	6–24 mg/dl
Creatinine	0.7	0.56–1.04 mg/dl
Sodium	138.3	135–145 mmol/l
Potassium	3.6	3.5–5 mmol/L
Calcium	2.5	2.2–2.6 mmol/L
INR	1	0.9–1.1
aPTT	24	25–36 s
TSH	0.03	0.03–3.5 mU/L^a^
Free T4	1.28	0.7–1.9 ng/dl
CA‐125	62.1	1.7–35 IU/ml
AFP	9.37	0–10 IU/ml
β‐hCG	45,926	7500–75,000 mIU/ml^b^

Abbreviations: AFP, alpha‐fetoprotein; aPTT, activated partial thromboplastin time; β‐hCG, beta‐human chorionic gonadotropin; CA‐125, cancer antigen 125; INR, International Normalized Ratio; T4, tetraiodothyronine; TSH, thyroid‐stimulating hormone.

^a^ Expected range in the second trimester of the pregnancy.

^b^ Expected range for 16–18 gestational weeks.

## 3 TREATMENT AND FOLLOW‐UP

After a thorough discussion of the findings, the patient opted for pregnancy termination. We started an inpatient analgesic therapy with oral misoprostol 100 μg every 12 h until cervix changes started. The induced labor progressed without complications and yielded a dead female fetus. The patient could be discharged home after improvement of the general condition. An expectant treatment of hyperreactio luteinalis with periodic follow‐up was arranged.

**FIGURE 2:**
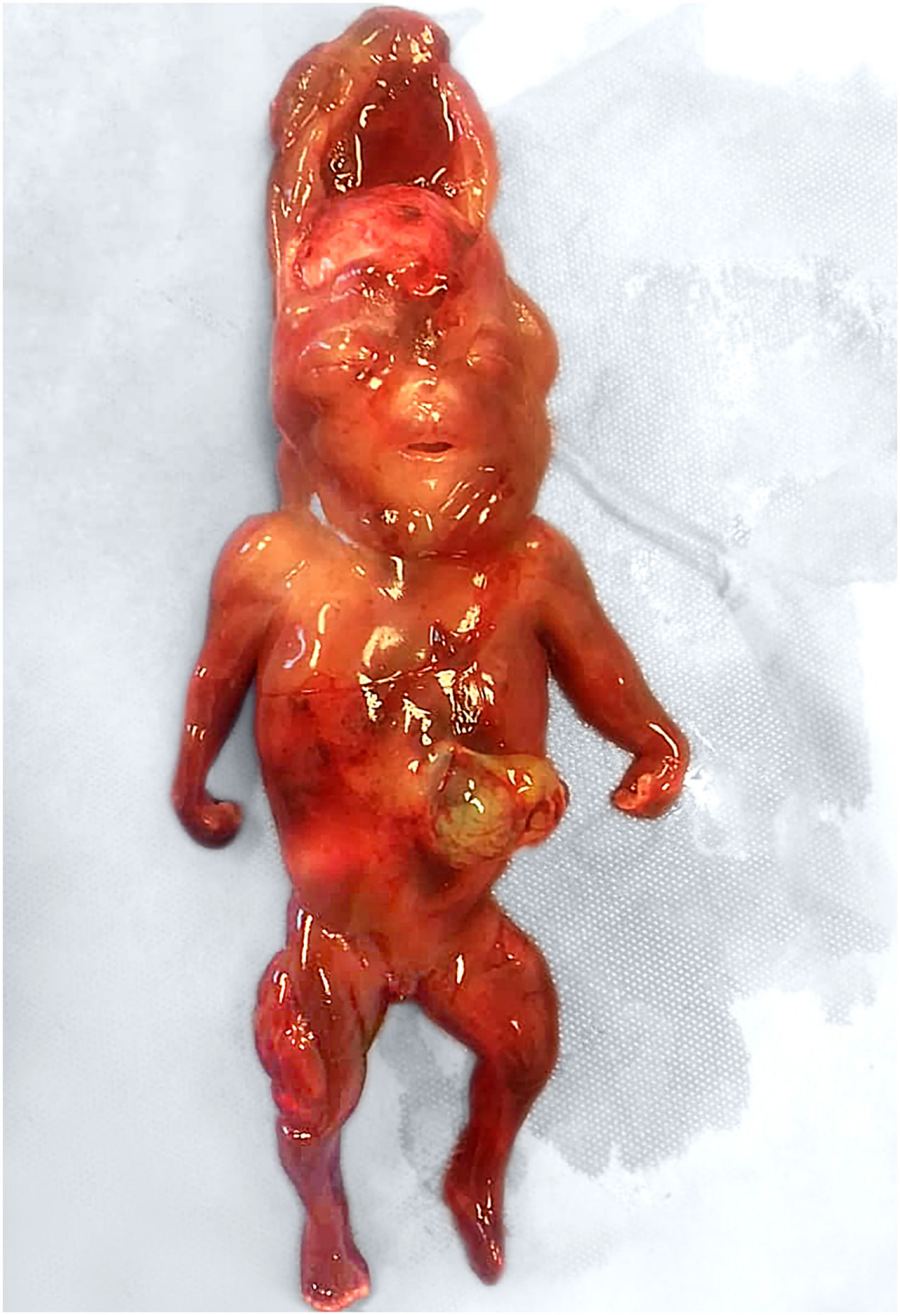
The female fetus. The cystic hygroma is opened frontally showing the exposed skull. A fluid collection fills the space between the scalp and skull and extends backward to the occipital region. Note the flexion deformity of the wrists and the muscular hypertrophy of the legs (more prominent on the right side).

The autopsy of the fetus showed a 4‐month‐female with a cephalic cystic hygroma and generalized muscular hypertrophy (Figure 2). There were no molar changes in the placenta. The parents refused the karyotyping of the fetus or placenta due to financial issues, as the testing is not covered with the treatment costs.

**FIGURE 3:**
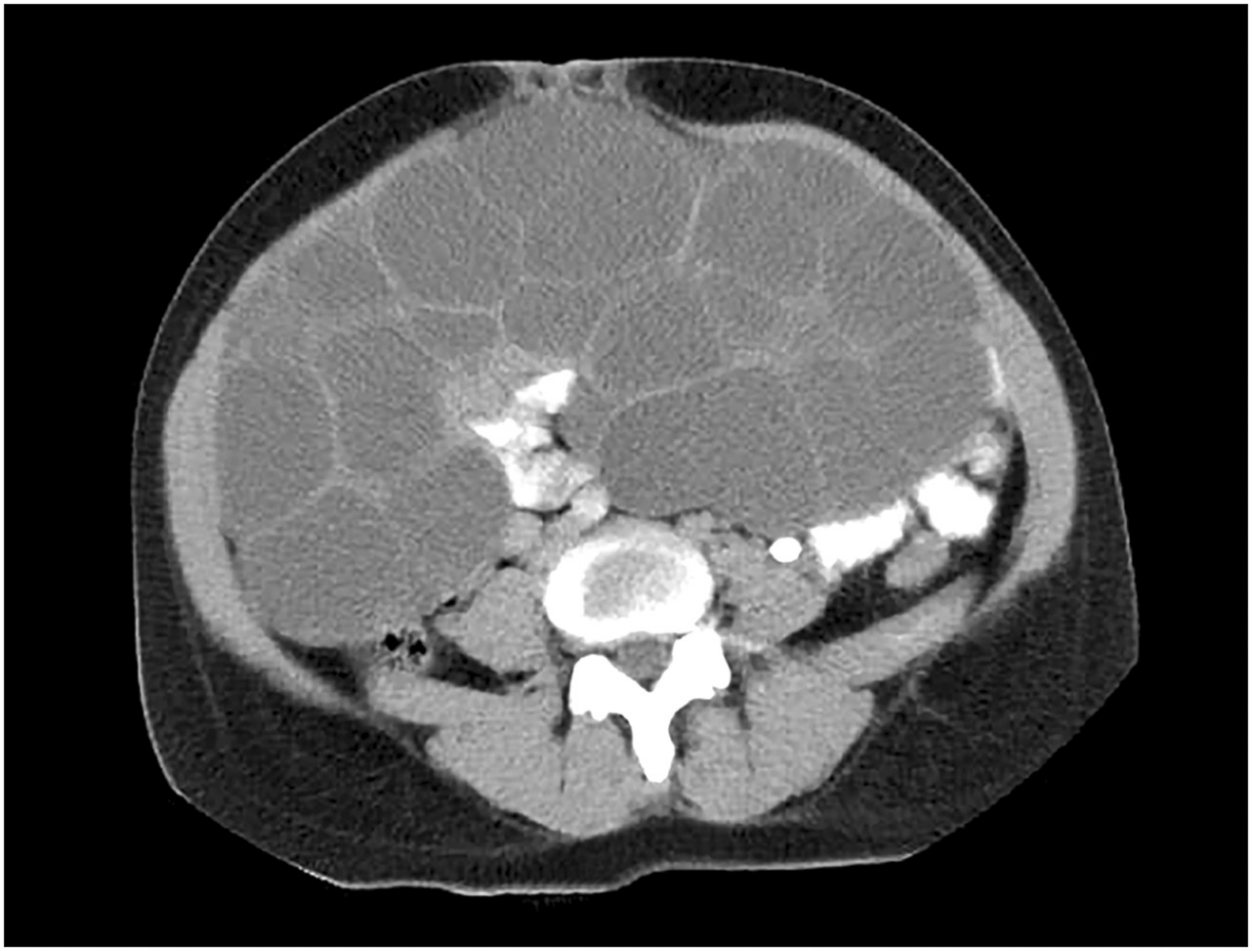
Axial view of the abdominal computed tomography in the venous phase with intravenous and oral contrast media showing a massive septated cystic complex of the ovaries that measures up to 40 × 15 × 23 cm. The structure fills the pelvis and extends to the epigastrium.

**FIGURE 4:**
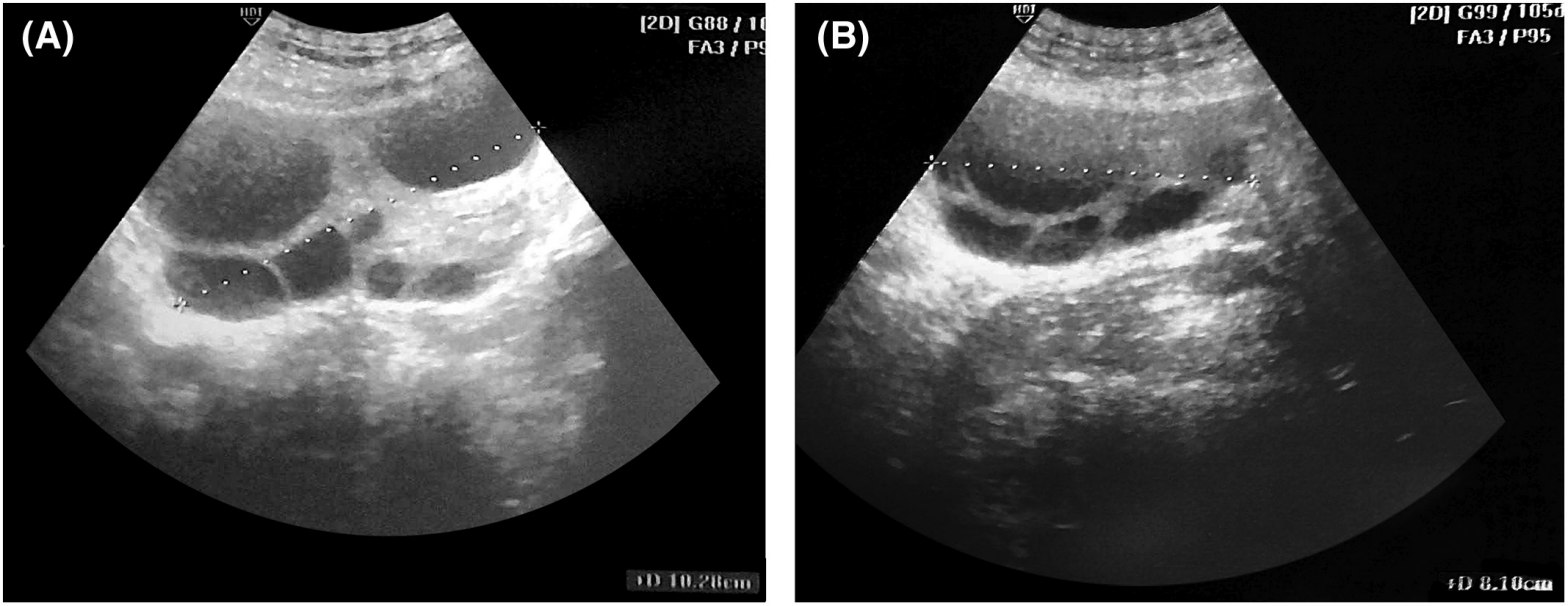
Ultrasound follow‐up eight weeks after delivery. The maximal diameter of the ovaries shrank to 10.3 cm on the right side (A) and 8.1 cm on the left side (B). The cystic complex shows an obvious regression in comparison with Figure 1B.

In the first follow‐up visit after one week, the patient was still complaining of abdominal discomfort and early satiety. The abdomen was still distended, and no regression of the cystic formation was noted on ultrasound. The computed tomography of the chest, abdomen, and pelvis after two weeks delineated the huge cystic complex that extended from the epigastrium to the pelvis and compromised the abdominal organs (Figure 3). No other pathologies were found. We decided to start hormonal therapy using an oral contraceptive preparation containing a combination of ethinylestradiol 0.03 mg and drospirenone 3 mg. Thereafter, the patient was followed every two weeks and the cysts showed gradual regression (Figure 4). Ten weeks after the termination of pregnancy, the cysts showed complete resolution and the patient was free of symptoms.

## 4 DISCUSSION

Cystic ovarian lesions of pregnancy can be neoplastic or functional, with the latter being the most common variant.^8^ Functional lesions can include polycystic ovarian syndrome, ovarian luteomas, and ovarian hyperstimulation syndrome, which is seen after ovarian induction for treatment of infertility.^3^ Hyperreactio luteinalis (HL) is considered one of the other less common causes of bilateral ovarian cystic formations in pregnancy.^2^

The exact cause of HL is still unclear.^3^ Many cases were related to the elevated β‐hCG levels, typically over 300,000 IU, in association with a molar pregnancy, choriocarcinoma, or even large placenta as in multiple gestations.^2^, ^4^, ^9^ However, as seen in this patient with spontaneous pregnancy and absence of previous ovarian stimulation, circulating β‐hCG levels can be normal in nearly 30% of patients.^3^ Therefore, HL can also develop in uncomplicated pregnancies. Suggested pathogenetic factors include ovarian hypersensitivity and the presence of hCG variants that have potent stimulatory effects.^3^ Under excessive stimulation, the ovarian granulosa and theca interna cells become hypertrophied and luteinized, which leads to the development of theca lutein cysts.^10^ A prior polycystic ovarian syndrome may predispose for HL during subsequent pregnancies.^1^

Hyperreactio luteinalis presents most commonly in the second or third trimester.^3^ Many cases are discovered incidentally, either with ultrasound or during cesarean section, whereas symptomatic cases usually present with abdominal discomfort or pain.^3^ Both hypothyroidism and hyperthyroidism were reported in patients with HL.^11^, ^12^ Patients with pre‐existing hypothyroidism show elevated levels of thyroid‐stimulating hormone (TSH), which can activate hCG receptors and induce secondary hyperreactio luteinalis.^12^ On the other hand, primary HL can be associated with TSH suppression due to the negative feedback effects of hCG on the pituitary‐thyroid axis.^13^ The laboratory results in our patient may indicate a slight degree of subclinical hyperthyroidism, as physiologic TSH levels in the first trimester of pregnancy can range from 0.03 to 2.5 mU/L.^11^ The tumor marker CA‐125 was slightly elevated in our patient (62.1, normal level up to 35 IU/ml). Due to ovarian cells hypertrophy, an elevation of CA‐125 can be anticipated in association with HL, and values up to 442 IU/ml were reported in the literature without indicating malignancy.^2^

Hyperreactio luteinalis can be associated with hyperandrogenism in 25%–30% of cases.^2^ This can cause masculinization or even virilization symptoms in the mother, but far less frequently in the fetus due to the presence of the placental barrier which converts testosterone to estrogen.^4^ In their review of 58 cases of HL, Malinowski et al.^3^ found only two cases of virilized female fetuses, with the presence of genital malformations requiring surgical correction. The female fetus in our case showed clear masculinization features with muscular hypertrophy, whereas no hyperandrogenic symptoms were noted in the mother. Other fetal effects may include intrauterine growth restriction and preterm delivery, which were reported in 32% and 38% of cases, respectively.^2^, ^3^

The association of fetal cystic hygroma with maternal HL was documented in only one case in the literature so far, with our case being the second one. Kalelioglu et al.^5^ described a 29‐year‐old woman in her first spontaneous pregnancy with a prenatal diagnosis of fetal cystic hygroma accompanying hyperreactio luteinalis. The female fetus showed clitoromegaly and bilateral pes equinovarus with normal chromosomal karyotype.

Cystic hygromas are congenital lymphatic malformations that can originate in different parts of the body, with the neck being the most common location.^6^ Half of the hygromas are associated with chromosomal abnormalities, which may warrant termination of pregnancy.^7^ Prenatal ultrasound can reveal the thin‐walled multiseptated collections. Interestingly, occipital lesions show a sonolucent region that consists of two symmetrical cavities separated by a midline septum,^7^ exactly as shown in Figure 1A.

One recent research by Yoshida et al.^14^ found that the overexpression of amphiregulin (an epidermal growth factor) is associated with accelerated proliferation of lymphatic endothelial cells, which plays a role in the pathogenesis of the cystic hygromas. If a correlation between HL and cystic hygroma is to be assumed, we may hypothesize that the hormonal imbalance seen in HL can affect lymphogenic growth factors in the fetus, hence inducing the formation of cystic hygroma. Of course, the concurrence may have arisen by chance in this case. Therefore, more research on this potential relationship is required.

Most cases of HL can be managed conservatively. Spontaneous regression is the rule, which occurs in the third trimester or within four to six months postpartum.^15^ Inpatient management, including analgesic therapy and fluid substitution, may be required.^16^ Ovarian suppression with oral contraceptives can be used to accelerate resolution,^1^ with surgical therapy being reserved for complicated cases with bleeding, torsion, or rupture.^17^ In our case, termination of pregnancy was requested by the parents due to the presence of cystic hygroma. Otherwise, the pregnancy can be allowed to progress, with vaginal delivery being the preferred route.^3^ Bishop et al.^16^ reported a patient with recurrence requiring treatment in two subsequent pregnancies. Therefore, vigilance surveillance is necessary for every patient with a previous history of hyperreactio luteinalis.

## 5 CONCLUSION

Although hyperreactio luteinalis is usually associated with ovarian hyperstimulation, it can develop in spontaneous pregnancies with normal gonadotropin levels. Conservative therapy is the rule, with the surgical intervention being reserved for complicated cases. Every prenatal ultrasound examination should include screening for cystic hygromas, as they may warrant termination of pregnancy. This report presented the second documented case of cystic hygroma associated with hyperreactio luteinalis. Clinicians are encouraged to report such cases to enable understanding of a probable causal relationship between the two entities.

## AUTHOR CONTRIBUTIONS

AG gathered the data, researched the literature, and wrote the first draft. DA treated and followed the patient and reviewed the article for scientific adequacy. MFS wrote the figure captions and edited the discussion. All authors reviewed and approved the final manuscript before submission.

## FUNDING INFORMATION

The authors received no funding regarding the publication of this article.

## CONFLICT OF INTEREST

The authors declare that there is no conflict of interest to be reported.

## CONSENT

Written informed consent was obtained from the patient to publish this report in accordance with the journal's patient consent policy.

## Data Availability

All data generated during this study can be accessed through direct communication with the corresponding author and the agreement of all research team members.
